# Prevalence and factors related to psychiatric symptoms in low risk pregnancy

**DOI:** 10.22088/cjim.11.2.211

**Published:** 2020

**Authors:** Mahbobeh Faramarzi, Farzan Kheirkhah, Shahnaz Barat, Pim Cuijpers, Elizabeth O'Connor, Reza Ghadimi, Karimollah Hajian-Tilaki, Zeynab Pahlavan, Angela Hamidia, Seyyedeh Mahboubeh Mirtabar, Mahtab Zeinalzadeh, Zahra Basirat

**Affiliations:** 1 Social Determinants of Health Research Center, Health Research Institute, Babol University of Medical Sciences, Babol, Iran,; 2 Infertility and Health Reproductive Research Center, Health Research Institute, Babol University of Medical Sciences, Babol, Iran; 3 Department of Clinical Neuro and Developmental Psychology, Amsterdam Public Health Research Institute, Vrije Universiteit Amsterdam, Amesterdam, Netherlands; 4 Center for Health Research, Kaiser Permanente Northwest, Portland, Oregon, USA; 5 Department of Epidemiology and Biostatics, Babol University of Medical Sciences, Babol, Iran,; 6 Clinical Research Development Unit of Ayatollah Rouhani Hospital, Babol University of Medical Sciences, Babol, Iran

**Keywords:** Psychiatric, Mental disorders, Pregnancy, Depression, Anxiety, Maternal distress

## Abstract

**Background:**

Psychiatric disorders are associated with poor pregnancy outcomes both for mother and child. This study aimed to determine the prevalence and related demographic risk factors of psychiatric symptoms among the pregnant women in Babol City.

**Methods:**

This cross-sectional study was conducted in five private and public obstetrics clinics of Babol city. During routine appointments of prenatal care, 176 pregnant women filled in three questionnaires including; sociodemographic questionnaire, Edinburg Prenatal Depression Scale (EPDS), and Symptom Checklist-25 (SCL-25). Wilcoxon test, Spearman correlation, and *multivariate logistic regression tests* were used to interpret the data.

**Results:**

The prevalence of depressive disorders was 15.4%% for Edinburg scores ≥13. The overall rate of maternal psychiatric symptoms (global severity index or GSI scores ≥ 1.75) was 48.5%. The prevalence of psychiatric symptoms was high; for 25% somatization, 258% anxiety, obsession-compulsion disorders or OCD 6.4%, 8.8% interpersonal sensitivity, 5.3% phobia, 7.6% paranoid ideation, and 1.2% psychoticism. Multivariate logistic regression revealed that pregnant women with history of abortion in previous pregnancy were at risk of depressive symptoms more (β=3.18, CI 1.28-7.93, p=0.01) than those without history of abortion. Also, the only demographic factor related to psychiatric symptoms was the age of pregnant women; younger age was associated with higher symptom levels for GSI ((r=-0.17).

**Conclusion:**

The high prevalence of psychiatric symptoms, especially depressive symptoms, in pregnant women highlights the need for continued research on screening, identifying the risk factors, and developing effective treatments for mental disorders in pregnant women.

Psychiatric disorders are one the most common morbidities of pregnancy that can have adverse effects on the mothers, child, and family (1). Depressive disorders are the most common psychiatric disorders with incidence of 1 out of 7–10 pregnant women (2). The prevalence of depression ranged 15% to 20% in low-and lower-middle-income countries (3). The prevalence of antenatal anxiety/depressive symptoms is reported 25% in 2014, in a general hospital in Babol city, North of Iran. (4). A recent review (2019) reported that the prevalence of antenatal depressed women with complicated pregnancies ranged from 12.5 to 44.2% (5). Similarly, the likelihood of high-risk pregnancy-related conditions such as gestational diabetes mellitus is higher in women with severe psychiatric disorders (20.9%) compared with women with non-psychiatric severe mental illnesses (8.3%) (6).

Psychiatric disorders during pregnancy are associated with poor quality of life for mothers (7). Also, depression/anxiety during pregnancy increases the risk of preterm birth, low birth weight, and intrauterine growth restriction (8-10). Antenatal psychiatric disorders increase the risk of psychological and developmental disturbances in children (11). Depressive disorders during pregnancy may be associated with poorer cognitive and behavioral function in children (11), attention disorders (13), emotional problems (14), childhood anxiety (13), and mother-child attachment disorders (15). Also, children of mothers with depression remain at increased risk of depression themselves into adolescence (16). 

Although a lot of researches have focused on prenatal and postnatal depression, increasing evidence shows substantial morbidity from other mental disorders, such as anxiety disorders, post-traumatic stress disorder (PTSD), and eating disorders (1). Two-thirds of pregnant women reported anxiety during pregnancy that was often comorbid with depressive disorders (17). A study reported the prevalence of PTSD 2.8% to 5.6% at around 6 weeks following birth (18).

To address these gaps in the literature, the study aimed to investigate to determine the prevalence of psychiatric symptoms among pregnant women in Babol city. The second purpose of the study was to investigate the possible association between psychiatric symptoms during pregnancy and the independent demographic factors. The current research is novel in a number of ways. First, there are limited data regarding the prevalence of psychiatric disorders in pregnant women in the world. Second, to the best of our knowledge, no published study has reported the prevalence of psychiatric symptoms in Iranian population. Finally, this theory is gaining popularity that factors associated with psychiatric disorders in pregnancy are related to specific cultural norms. Thus, Iranian culture influences the way pregnant women expose to psychiatric disorders. Therefore, an understanding of factors valued by Iranian culture will increase the success of potential interventions to prevent psychiatric disorders.

## Method

**Study participants: **This is a descriptive cross-sectional study of pregnant women in the maternity care of Babol city (North of Iran) from May to September 2019. This research was approved by the Ethics Committee of National Institute for Medical Research Development (IR.NIMAD.REC.1398.015). 

A convenience sample of participants was collected in five obstetrics clinics of Babol city (a large public hospital and four private clinics). With the assumption of the prevalence of depressive symptoms in 20% in pregnant women, this allocated sample size estimated 170, in which the marginal error of estimate does not exceed than 0.06 with 95% confidence level.

Pregnant women were eligibile to participate in the study, if they met the following criteria: age 18 years or older, having at least a primary school education, no severe cognitive or developmental impairment, no pregnancy complication, and consent to enter the study. Women having history of high risk pregnancy such as hypertension, hemorrhage, and diabetes were excluded. Of the 200 women invited to join the study, 20 did not meet the criteria to enter the study or declined. Also, 4 persons who did not fill out the questionnaires completely were excluded from the analysis. Therefore, the data of 176 pregnant women were analyzed. 

 During routine prenatal care appointments, a midwife explained the study and invited the pregnant women to enter the study. Then she assessed the inclusion/exclusion criteria for the women who accepted her invitation. At the end of the appointment, the midwife gave the participant the questionnaires to be completed. All of the participants filled three questionnaires including; sociodemographic questionnaire, Edinburg Prenatal Depression Scale (EPDS), and Symptom Checklist-25 (SCL-25). 

**Measurements: **A sociodemographic questionnaire was used to describe the characteristics of the participants in terms of age, educational level, marital status, job, parity. The use of the Edinburg Depression Scale (EPDS) as a component of universal routine assessment to identify perinatal depression is one of the key recommendations of the worldwide research (19). EPDS is a 10-item questionnaire and a widely used instrument to assess depressive symptoms in both the antenatal and postnatal periods. We used an EPDS cutoff ≥13 (“possible” depression) to screen positive for depression symptoms (19). We used validated Persian EPDS in this study (20). Symptom Checklist-25 (SCL-25) is a brief form of SCL-90 with 25 questions in a Likert 0-4 included: never (0), a few (1), somewhat (2), great (3) and very great or severe (4). The scale covers eight subscales including somatization, obsession-compulsion disorders (OCD), interpersonal sensitivity, phobia, depression, anxiety, paranoid ideation, and psychoticism. Raw scores were calculated by dividing the sum of scores for each subscale by the number of items. Also, the global severity index (GSI) was used to measures the extent or depth of the individual’s mental health problems; by dividing the sum of scores of all questions on the number of questions. We used the Iranian version of SCL-25 that has suitable validity (Cronbach’s alpha 0.97) and reliability (re-test coefficients 0.78) (21). The current study used a cutoff SCL-25 ≥1.75 to indicate the likely mental health problems for each of the SCL-25 subscales and for GSI (22). 

**Statistical analysis:** All data were analyzed using SPSS (Version 17.0, IBM, Chicago, IL, USA) software. Mothers’ demographic characteristics were categorized as follows: age (18-29 vs >30), education (below diploma, high school graduate or higher), employment (employed, un employed), place of living (urban, rural), gestational age (<20, 20-41 weeks), parity (0, 1, ≥2), and previous abortions (0, ≥1). All subscales of SCL-25, and GSI) were not normally distributed so we conducted Wilcoxon rank-sum tests to compare the mean mental health scale scores across demographic subpopulations. To assess whether the likelihood of meeting the EPDS cut-off indicating possible depression differed by demographic characteristics, stepwise multivariate logistic regression test were used. Statistical significance was determined as a p<0.05, all P values were two-tailed.

## Results

Table 1 describes the demographic characteristics of the participants. The mean age of the subjects was 29.05±5.74 years. A large proportion of the women had high school/university level of education (89.8%) and 78.4% were unemployed. 

The prevalence of depressive symptoms (based on EPDS scores≥13) was 15.4%. The mean (SD) EPDS scores was 8.16(4.10). The mean GSI score was 1.99±1.31. The prevalence of having psychiatric symptoms (based on GSI≥1.75) was 49.5%. The results of this study showed (based on the mean score each subscale SCL-25 ≥1.75) a rate of somatization at 25%, OCD at 6.4%, interpersonal sensitivity at 8.8%, phobia at 5.3%, depression at 23.4%, anxiety at 25.8%, paranoid ideation at 7.6%, and psychotism at 1.2%. 

**Table1:** Characteristics of the study population

**Variables**	**N (%)**
Age (years)	
18-30 ≥30	92 (52.3) 84 (41.1)
Education	
Below diploma High school graduate University level	18 (10.2) 82 (46.6) 76 (43.2)
Job	
Employee Unemployed	38 (21.6) 138 (78.4)
Gestational Age (Weeks)	
<20 20-41	39 (22.2) 110 (62.5)
Place of Living	
Town Village	119 (67.6) 57 (32.4)
Parity	
0 1 ≥2	101 (57.4) 60 (34.1) 15 (8.5)
Number of Abortion	
0 ≥1	135 (76.7) 41 (23.3)

To determine whether demographic characteristics’ population were associated with the risk of psychiatric symptoms, we analyzed the mean scores of the SCL-25 subscales based on categorical demographic of the participants (table 2). The mean scores were higher for women under 30 years old for anxiety (p=0.05) and psychoticism (p=0.05). The mean scores of phobic anxiety and paranoid ideation were higher for women with a diploma than for those with less education, and intermediate for those who attended university (p=0.04 for phobic anxiety, p=0.02 for paranoid ideation). 

The mean scores of other subscales of SCL-25, as well as GSI did not differ among the categorical groups for other demographic characteristics (urban women vs rural, gestational age of ≤20 weeks vs 21-41 weeks; parity one child vs more than one child, with vs. without a history of abortion). Also, the only demographic factor related to psychiatric symptoms was the age of pregnant women; younger age was associated with higher symptom level for GSI ((r=-0.17). We did not find an association between the likelihood of meeting the cutoff for depressive symptoms (EPDS ≥13) and any demographic characteristics except for abortion. Pregnant women with history of abortion in previous pregnancy were at risk of depressive symptoms more (β=3.18, CI 1.28-7.93, p=0.01) than those without history of abortion (table 3). 

**Table 2:** Comparison of mean (SD) scores of the psychiatric symptoms according to characteristics of the study Population

**Variables**	**Somatization** **Mean(SD) ** ***P-value	**Interpersonal** **Mean(SD) ** **P-value**	OCD* **Mean(SD) ** **P-value**	**Depression** **Mean(SD) ** **P-value**	**Anxiety** **Mean(SD) ** **P-value**	**Phobic Anxiety** **Mean(SD) ** **P-value**	**Paranoid Ideation** **Mean(SD) ** **P-value**	**Psychoticism** **Mean(SD) ** **P-value**	GSI** **Mean(SD) ** **P-value**
Age (years) 18-30 ≥30	1.15 (0.81) 1.05 (0.65) 0.580	0.79 (0.72) 0.60 (0.62) 0.064	0.75(0.73) 0.60(0.59) 0.208	0.74 (0.73) 0.61 (0.56) 0.378	0.88(0.81) 0.64(0.65) 0.050	0.48(0.55) 0.42(0.50) 0.506	0.83(1.04) 0.57(0.79) 0.139	0.35 (0.39) 0.25 (0.38) 0.048	2.16(1.42) 1.80(1.13) 0.113
Education Under diploma Diploma university	0.86 (0.61) 1.18 (0.79) 1.08 (0.69) 0.136	0.80 (0.66) 0.68 (0.62) 0.69 (0.74) 0.495	0.56(0.59) 0.68(0.58) 0.71(0.78) 0.434	0.73 (0.73) 0.68 (0.59) 0.67 (0.72) 0.996	0.64(0.58) 0.85(0.79) 0.71(0.73) 0.451	0.23(0.42) 0.50(0.54) 0.46(0.52) 0.036	0.35(0.49) 0.97(1.05) 0.51(0.81) 0.025	0.35 (0.44) 0.37 (0.38) 0.23 (0.37) 0.590	1.63(1.09) 2.14(1.34) 1.92(1.30) 0.164
Job Employee Un employee	1.13 (0.71) 1.09 (0.75) 0.710	0.77 (0.81) 0.68 (0.64) 0.839	0.73(0.90) 0.67(0.60) 0.576	0.79 (0.88) 0.65 (0.59) 0.766	0.83(0.77) 0.75(0.74) 0.577	0.56(0.58) 0.42(0.51) 0.148	0.69(0.92) 0.71(0.95) 0.955	0.36 (0.49) 0.29 (0.36) 0.715	2.16(1.54) 1.95(1.24) 0.705
Gestational Age (Weeks) <20 20-41	1.09 (0.71) 1.10 (0.75) 0.996	0.45 (0.48) 0.81 (0.74) 0.008	0.58(0.62) 0.75(0.71) 0.185	0.52 (0.55) 0.75 (0.69) 0.078	0.62(0.53) 0.78(0.79) 0.553	0.48(0.60) 0.42(0.48) 0.735	0.40(0.64) 0.76(0.98) 0.057	0.27 (0.29) 0.31 (0.40) 0.882	1.77(1.18) 2.04(1.34) 0.354
Place of Living Town Village	1.13 (0.79) 1.04 (0.61) 0.943	0.72 (0.70) 0.66 (0.63) 0.749	0.66(0.65) 0.73(0.72) 0.583	0.67 (0.65) 0.70 (0.70) 0.967	0.75(0.76) 0.82(0.73) 0.326	0.43(0.50) 0.50(0.58) 0.548	0.68(0.93) 0.78 0.390	0.29 (0.40) 0.35 0.113	1.98(1.35) 2.03(1.21) 0.509
Parity 0 1 2 ≥	1.09 (0.75) 1.16 (0.68) 0.96 (0.84) 0.165	0.70 (0.64) 0.75 (0.74) 0.50 (0.71) 0.117	0.72(0.68) 0.67(0.69) 0.45(0.54) 0.295	0.71 (0.70) 0.68 (0.60) 0.53 (0.63) 0.337	0.80(0.74) 0.71(0.76) 0.76(0.79) 0.822	0.46(0.56) 0.46(0.48) 0.35(0.46) 0.490	0.67(0.87) 0.75(1.04) 0.78(0.97) 0.784	0.35 (0.40) 0.23 (0.29) 0.33 (0.55) 0.851	2.02(1.27) 2.02(1.27) 1.68(1.67) 0.172
Number of Abortion 0 ≥1	1.09 (0.70) 1.14 (0.85) 0.935	0.73 (0.68) 0.58 (0.66) 0.069	0.72(0.71) 0.54(0.52) 0.231	0.70 (0.69) 0.62 (0.55) 0.702	0.77(0.74) 0.76(0.79) 0.618	0.47(0.53) 0.38(0.51) 0.249	0.73(0.93) 0.64(0.98) 0.400	0.31 (0.37) 0.29 (0.44) 0.300	2.03(1.27) 1.88(1.44) 0.310

*Obsession Compulsion Disorder

** Global Severity of Symptoms Index

*** Results of Wilcoxon tests

**Table 3:** The relationship between demographic factors and depressive symptoms based on stepwise multiple logistic regression

**variables**	**No depressive symptoms** **N (%)**	Depressive symptoms* **N (%)**	**Odds ratio** **(95% CI** **(**	**p-value
Age (years) 18-30 ≥30	76 (82.6) 72 (86.7)	16 (17.4) 11 (13.3)	Ref 0.63 (0.22, 1.82)	0.39
Education Under diploma Diploma university	13 (76.5) 66 (80.5) 69 (90.8)	4 (23.5) 16 (19.5) 7 (9.20)	Ref 0.80 (0.20, 3.20) 0.26 (0.53, 1.31)	0.75
Job unemployed Employe	35 (92.1) 113 (82.5)	3 (7.9) 24 (17.5)	Ref 0.78 (0.18, 3.30)	0.74
Gestational Age (Weeks) <20 20-41	34 (87. 2) 90 (82.6)	5.0 (12.8) 19 (17.4)	Ref 1.50 (0.47, 4.76)	0.48
Place of Living Urban Rural	98 (83.1) 50 (87.7)	20 (16.9) 7.0 (12.3)	Ref 0.56 (0.18, 1.70)	0.30
Parity 0 1 ≥2	88 (87.1) 48 (80.0) 12 (85.7)	13 (12.9) 12 (20) 2 (14.3)	Ref 0.94 (0.28, 3.19) 0.74 (0.11, 4.81)	0.92
Number of Abortion 0 ≥1	119 (88.8) 29 (70.7)	15 (11.2) 12 (29.3)	Ref 3.62 (1.81, 11.11)	0.024*

*Depressive symptoms defined as Edinburg depression score≥13, No depressive symptoms: Edinburg depression score<13

**Results of stepwise multivariate logistic regression

## Discussion

This is the first study to examine the prevalence and demographic relations of psychiatric symptoms in women with low risk pregnancies in Babol City. We found that the prevalence of depressive symptoms was high (15.7%). The percentage of depressive symptoms found in this study was lower than in other studies. The prevalence of depression in pregnancy may vary from 17.5% in Asia, 19.5% in Western Europe (23), and 33% in the United States (24). The rate of depressive symptoms ranged 15% to 28% in Brazil with the Hospital Anxiety and Depression Scale (HADS) to assess depression (25). However, the prevalence of depression in some studies was reported higher than this study. More than half of pregnant women suffered from depression in Pakistan (26). The differences of prevalence may be related to type of study, the diversity of assessment tools, diversity of cutoff points of depression tools, and sociocultural heterogeneity of nations (27). The rate of maternal severity psychiatric symptoms (GSI of SCL-25≥1.75) was high (49.5%) with the mean GSI score 1.99±1.31 in women with low risk pregnancies of Babol City. A study reported the GSI 1.20±0.28 for pregnant women in Shanghai based on SCL-90-R (28). While we cannot determine the proportion with psychiatric illness based on the GSI cutoff, it may be higher than the rates in the USA, where a study found the 25.3% of women met DSM-IV criteria for a psychiatric disorder (29). A study identified that 37.14% of pregnant women needed to psychiatric intervention in Iran- Kashan City (30).

The only demographic factor related to psychiatric symptoms was the age of pregnant women. From this study, it also appears that there is no association between psychiatric symptoms and education, parity, location of living, and job of pregnant women. This finding is not supported by the previous study by Mayberry et al. who noted that the young maternal age, low education, not being employed full time, and multiparty were risk factors for depressive symptoms (31). Also, another study reported that maternal low educational attainment increased the risk of psychiatric symptoms in antenatal period (32). Culture-specific factors may be contributing to differences in risk factors of antenatal psychiatric disorders.

The only demographic factor predicted to depressive symptoms was the history of previous abortion. From this study, women with history of abortion were at 3.18 more at risk of depressive symptoms. This finding is supported by the previous research that emphasized pregnant women with a history of spontaneous abortion are at risk of psychiatric morbidities in subsequent pregnancies (33).

There are some limitations that may affect the generalization of the results. First, this is conducted in one city and may not be generalized and that the sample size is relatively small. Second, the assessment of psychiatric symptoms was performed with self-report scales. It is suggested that in future studies clinical interviews with a specialist may be added in the psychological scales. Second, we did not report the rate of psychiatric symptoms in the postnatal period. We recommend future studies to measure the prevalence of psychiatric disorders from prenatal to delivery and postnatal period. Further, this study was conducted on women with low risk pregnancies, so it would not be generalized to high risk pregnancies. Complicated pregnancies may increase the prevalence of psychiatric disorders in antenatal period. In the future, it is recommended to assess the prevalence and risk factors of psychiatric disorders in women with high risk pregnancy. 

The high prevalence of psychiatric symptoms, especially depressive symptoms, in low risk pregnant women emphasize the need for continued research to screen, identify the risk factors and develop effective treatments for psychiatric disorders in pregnant women. Our findings suggest that the detection of psychiatric disorders during pregnancy may be valuable in helping women access the needed mental health services. The results suggest that healthcare/ physicians should be aware of about one third of women are at risk of depression. Therefore, psychiatric symptom screening during routine antenatal care may improve the detection of psychiatric disorders. Development and testing of safe treatments for pregnant women may increase the rates of treatment-seeking of those who were diagnosed as psychiatric disorders. 
